# Step-by-step quantitative analysis of focal adhesions

**DOI:** 10.1016/j.mex.2014.06.004

**Published:** 2014-07-07

**Authors:** Utku Horzum, Berrin Ozdil, Devrim Pesen-Okvur

**Affiliations:** Izmir Institute of Technology, Department of Molecular Biology and Genetics, 35430 Izmir, Turkey

**Keywords:** Focal adhesion, Particle analysis, Image processing, ImageJ

## Abstract

Focal adhesions (FAs) are specialized adhesive structures which serve as cellular communication units between cells and the surrounding extracellular matrix. FAs are involved in signal transduction and actin cytoskeleton organization. FAs mediate cell adhesion, which is a critical phenomenon in cancer research. Since cells can form many and micrometer scale FAs, their quantitative analysis demands well-optimized image analysis approaches [1–3]. Here, we have optimized the analysis of FAs of MDA-MB-231 breast cancer cells. The optimization is based on proper processing of immunofluorescence images of vinculin, which is one of the markers of FAs. All image processing steps are carried out using the ImageJ software, which is freely available and in the public domain. The advantages of our method are:

• The analysis steps are simplified by combining different plugins of the ImageJ program.
• FAs are better detected with minimal false negatives due to optimized processing of fluorescent images.
• This approach can be applied to quantify a variety of fluorescent images comprising focal and/or localized signals within a high background such as FAs, one of the many complex signaling structures in a cell.

## Graphical abstract

**Figure:**
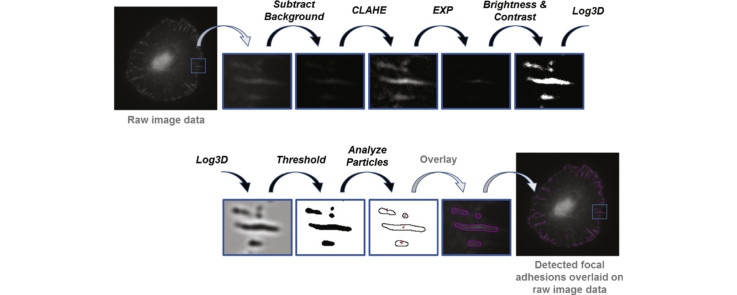

**Materials**

• Fluorescence images
• ImageJ software

**Critical points**

- Sample should be properly immunostained so that good quality fluorescence images can be captured.
- The images should be of high quality; unfocused images and inadequate resolution hinder the quality of the analysis.
- Fluorescence images should ideally be captured at 100× magnification.
- If color images are captured, single channel images should first be created with the ImageJ split channel command.
- Use raw image data which are greyscale images without any special preprocessing.

## Method details

Here, we have analyzed focal adhesions of MDA-MB-231 cells. However, our method can be applied to not only any cell type such as fibroblasts, endothelial cells or leucocytes but also to any structure in the cell with a localized signal such as focal adhesions, endosomes or invadopodia. Focal adhesions were immunostained for vinculin using a vinculin specific primary antibody (Sigma Cat no: V9131), followed by a fluorophore conjugated secondary antibody. Other markers of focal adhesions such as paxillin or focal adhesion kinase could also be easily used for analysis. 8-bit images were captured using an Olympus epi-fluorescence microscope with an Olympus infinity corrected 100× oil UPlanSApo objective with a numerical aperture of 1.4 and an Olympus SLR E-330 camera such that each image was 3136 pixels by 2352 pixels and 37 pixels corresponded to 2 μm. All steps of image processing were carried out using ImageJ which is freely available and in the public domain. ImageJ provides a wide range of processing and analysis approaches possible via not only built-in functions but also numerous plugins. ImageJ has been in use for some time by researchers. However, choosing the right combination and order of processing steps is crucial for obtaining meaningful quantitative results.

The image processing optimized here aims to identify FAs immunostained for vinculin. The raw fluorescent images are processed in steps as follows:

• Step 1: Apply subtract background. Selection of sliding paraboloid option changes the rolling ball to a parabolic with the same ball radius in pixels but with sharper curvature. A parabola slides in different directions over the image and calculates and subtracts local background from the original image. We chose the sliding paraboloid option with the rolling ball radius set to 50 pixels.
• Step 2: Enhance the local contrast of the image by running clahe (Contrast Limited Adaptive Histogram Equalization). The plugin clahe offers three critical parameters which are block size, histogram bins and maximum slope to clarify desired objects. We used the following values: block size = 19, histogram bins = 256, maximum slope = 6, no mask and fast [4].
• Step 3: Apply mathematical exponential (exp) to further minimize the background.
• Step 4: Adjust brightness & contrast automatically. The brightness & contrast tool updates the lookup table of the image based on an analysis of the histogram of the image.
• Step 5: Run Log3D (Laplacian of Gaussian or Mexican Hat) filter. The log3D plugin filters the image based on user predefined parameters which are standard deviations in *X*, *Y* and *Z* directions. For 2D images *Z* direction is not relevant and slice per slice processing is used. We defined the size of log3D filter as sigma *X* = 5 and sigma *Y* = 5 [5].
• Step 6: Run the threshold command. The threshold command converts a grayscale image to a binary image with two pixel values, 255 (white) and 0 (black). For the threshold command, there are options such as Huang, triangle, mean or default. In addition, the user can adjust minimum and maximum threshold values or set the threshold levels according to an analysis of the histogram of the current image automatically. Here we used the default method and threshold was adjusted automatically.
• Step 7: Execute analyze particles command. The analyze particles command scans the thresholded (binary) image and finds the edges of objects or particles. Essentially, particle analyzer counts and measures particles according to user pre-defined parameters which are size and circularity. We set these parameters as follows: size = 50-infinity and circularity = 0.00–0.99.

As a result of the analyze particles process, outlines of the particles found can be saved and overlaid on the original image to visually confirm faithful detection of FAs.

Our analysis on FAs of MDA-MB-231 cells cultured on fibronectin, an extracellular matrix protein that promotes cell adhesion and on K-casein, a blocking protein that prevents cell adhesion, determined the following [6]:

**Table:** 

	Number of FAs per cell	Area of FA	Total area of FAs per cell
Fibronectin	65 ± 14	0.56 ± 0.02 μm^2^	36.09 ± 8.45 μm^2^
K-casein	5 ± 1	0.38 ± 0.04 μm^2^	2.04 ± 0.63 μm^2^

The data is presented as average ± standard error. Number of FAs per cell = Total number of FAs identified in one cell. Area of FA = Area of a single FA. Total area of FAs per cell = The area of all FAs in one cell are summed up. Number of cells analyzed = 6–9 number of FAs analyzed = 0–348.

As expected, cells form more and larger FAs on a fibronectin coated surface compared with a K-casein coated surface (*p* < 0.05).

## Additional information

### Background information

Focal adhesions (FAs) are multifunctional structures comprising over a hundred proteins. FAs provide not only physical contact between a cell and its extracellular environment but also mediate inside-out and outside-in signaling [7]. Therefore, quantitative analysis of FAs is crucial for understanding important cellular phenomena such as cell morphology, motility, tissue organization, growth and differentiation [8,9]. Since cells can form many and micrometer scale FAs, their quantitative analysis demands well-optimized image analysis approaches [1–3]. The dimensions of focal adhesions range from 0.25 to 10 μm depending on the maturation stage of the FA as well as the cell type. Mature focal adhesions are also associated with the cytoskeleton [2,10]. There can be more than a hundred proteins associated with a focal adhesion and the identification of focal adhesion is possible by labeling protein of interest within this complex. Labeling of a mechanical stabilizer protein, vinculin, is the best suitable method technically and commercially [10].

### Useful links

ImageJ software: http://rsbweb.nih.gov/ij/download.html
Log3D plugin: http://bigwww.epfl.ch/sage/soft/LoG3D/
CLAHE plugin: http://rsbweb.nih.gov/ij/plugins/clahe/index.html
Similar image processing package: http://fiji.sc/Fiji
